# Supplementary figures and images for: The mechanism of dynamic steady states in lamellipodia
Source: PLoS Comput Biol. 2025 Oct 7;21(10):e1013572. doi: 10.1371/journal.pcbi.1013572 (PMC12517506; doi:10.1371/journal.pcbi.1013572)

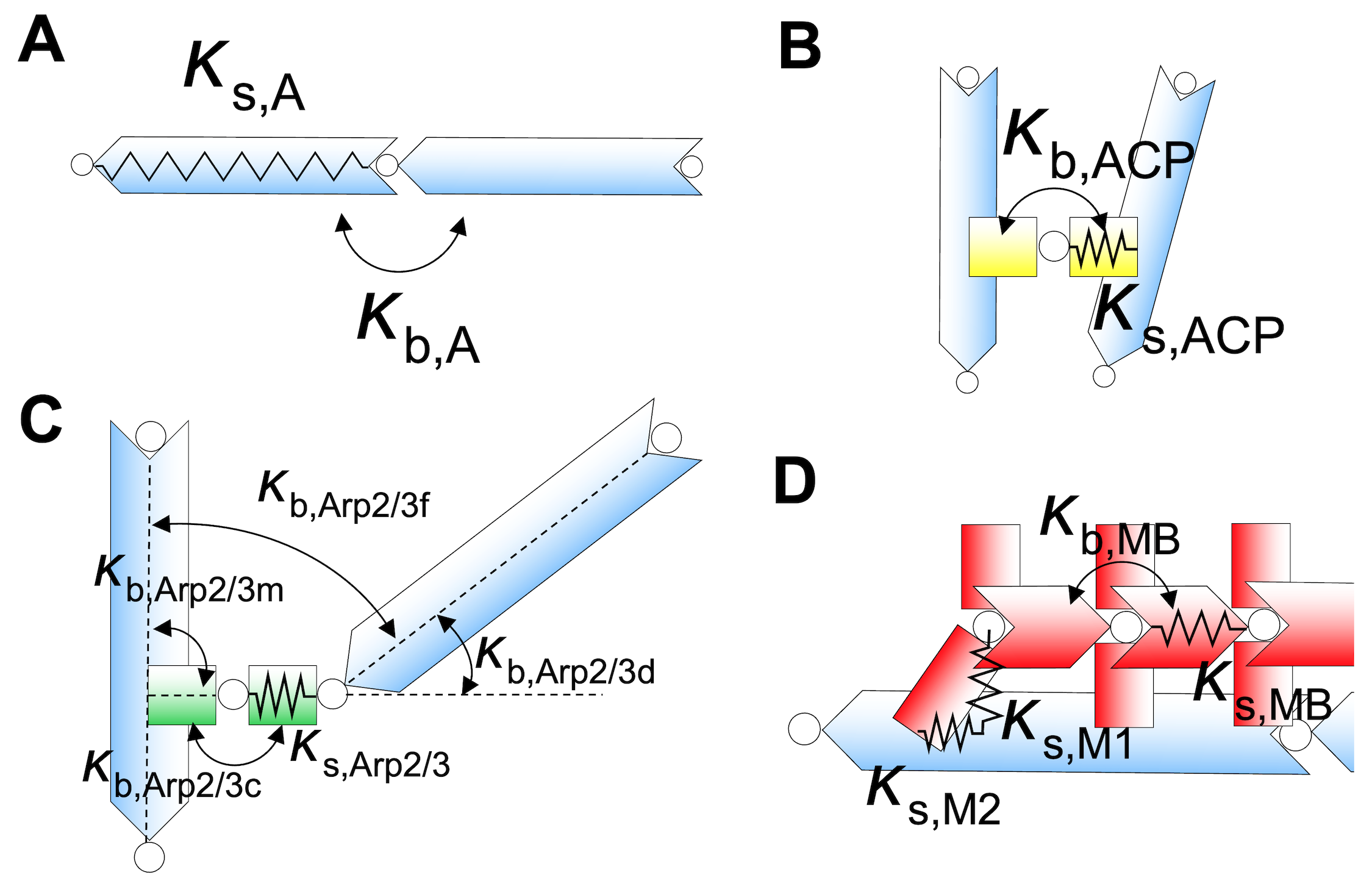

Supplement: S1 Fig — (A) F-actin, (B) ACP, (C) Arp2/3 complex, and (D) motor. κb and κs indicate bending and extensional stiffnesses that maintain equilibrium angles and lengths, respectively. In (C), the torsional stiffness is not included in the schematic. Detailed descriptions about these stiffness parameters and their values are written in S1 Table. (TIFF) [file pcbi.1013572.s001.tiff]

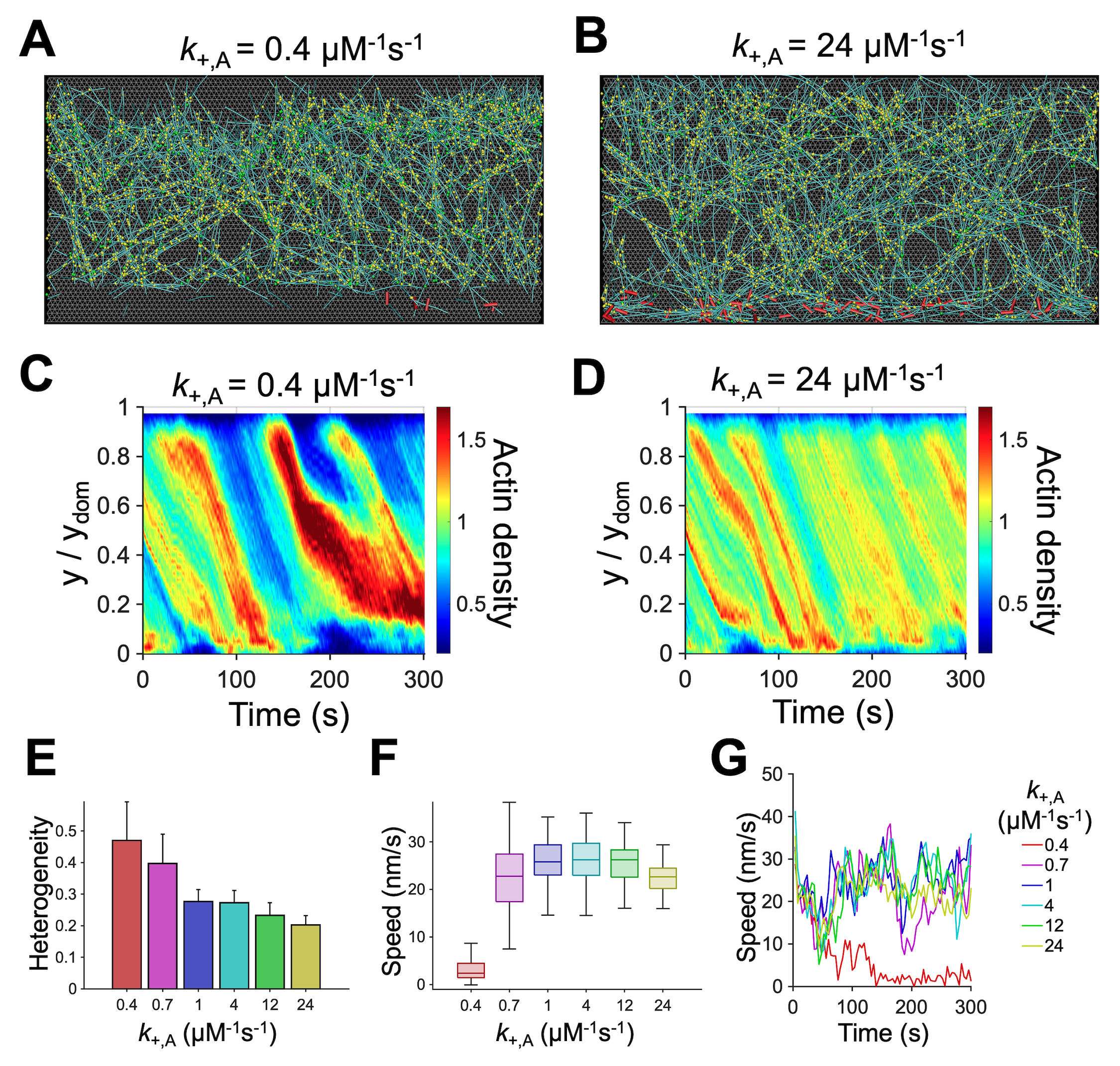

Supplement: S2 Fig — (A, B) Snapshots of the branched network taken at ~150 s with a lower (0.4 µM-1s-1) or higher (24 µM-1s-1) polymerization rate constant (k+,A) than that of the reference case, 12 µM-1s-1. (C, D) Kymographs of actin concentration as a function of y position and time with different k+,A. With lower k+,A, network heterogeneity increased because the network was contracted toward the -y boundary before sufficient F-actins were assembled near the + y boundary. (E) Heterogeneity of the network quantified as a coefficient of variation in actin density in the y direction. The network was more heterogeneous with deficient actin polymerization. (F) Retrograde flow speed depending on k+,A. With deficient actin polymerization, flow speed is lower, and the network failed to reach steady state. (G) Time evolution of retrograde flow speed. (TIFF) [file pcbi.1013572.s002.tiff]

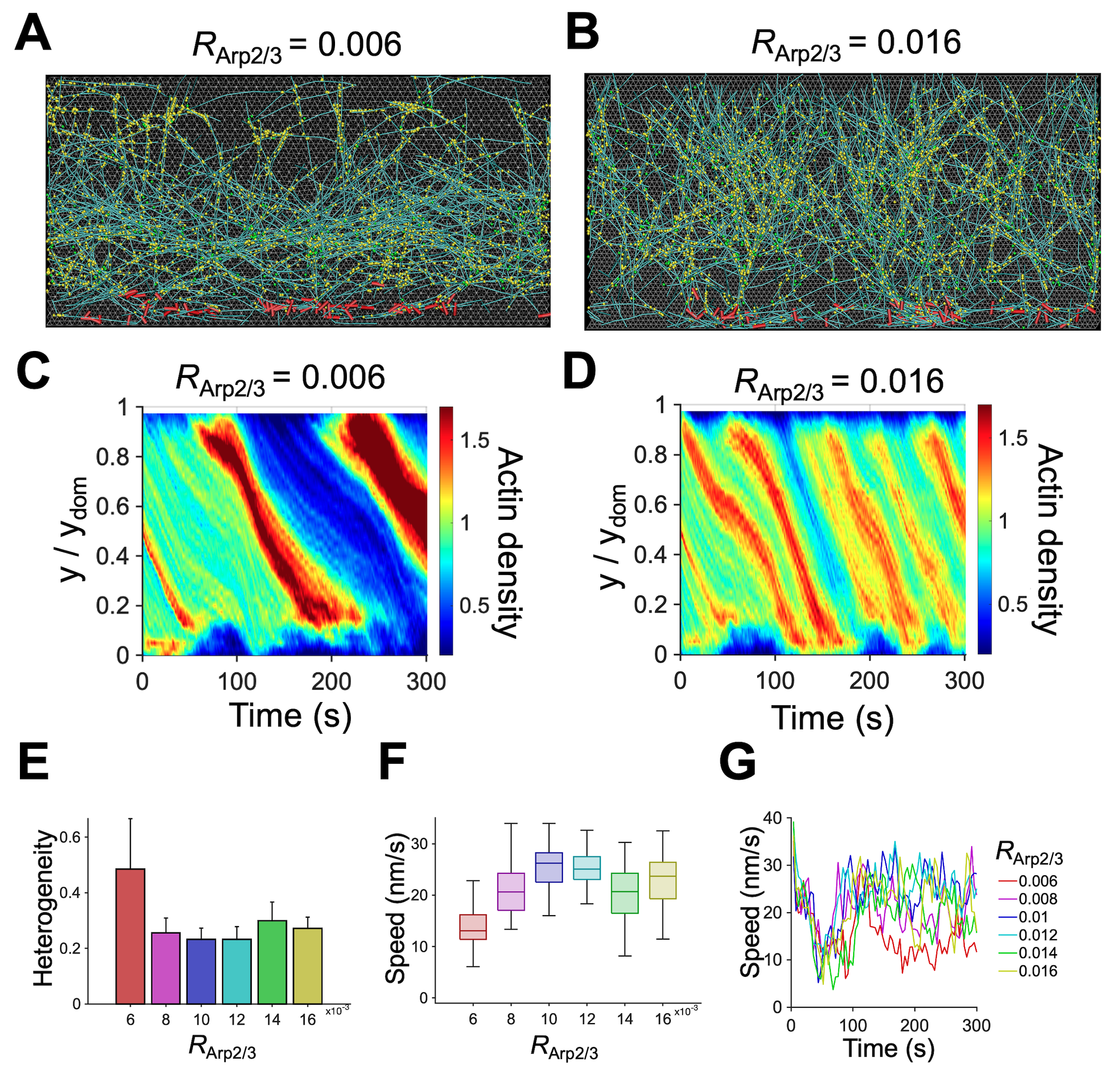

Supplement: S3 Fig — (A, B) Snapshots of the branched network taken at ~150 s with a lower (0.006) or higher (0.016) Arp2/3 density (RArp2/3) relative to that of the reference condition, 0.01. (C, D) Kymographs of actin concentration as a function of y position and time with different RArp2/3. With low RArp2/3, the network could not grow sufficiently, leading to higher network heterogeneity and lack of continuity in the y direction. By contrast, high RArp2/3 resulted in the formation of more branches on a fraction of vertically growing structures, so the network became more heterogeneous with lower connectivity in the x direction. (E) Heterogeneity of the network quantified as a coefficient of variation in actin density in the y direction. (F) Retrograde flow speed with different RArp2/3. A decrease in the flow speed was noticeable at RArp2/3 < 0.01. (G) Time evolution of retrograde flow speed. (TIFF) [file pcbi.1013572.s003.tiff]

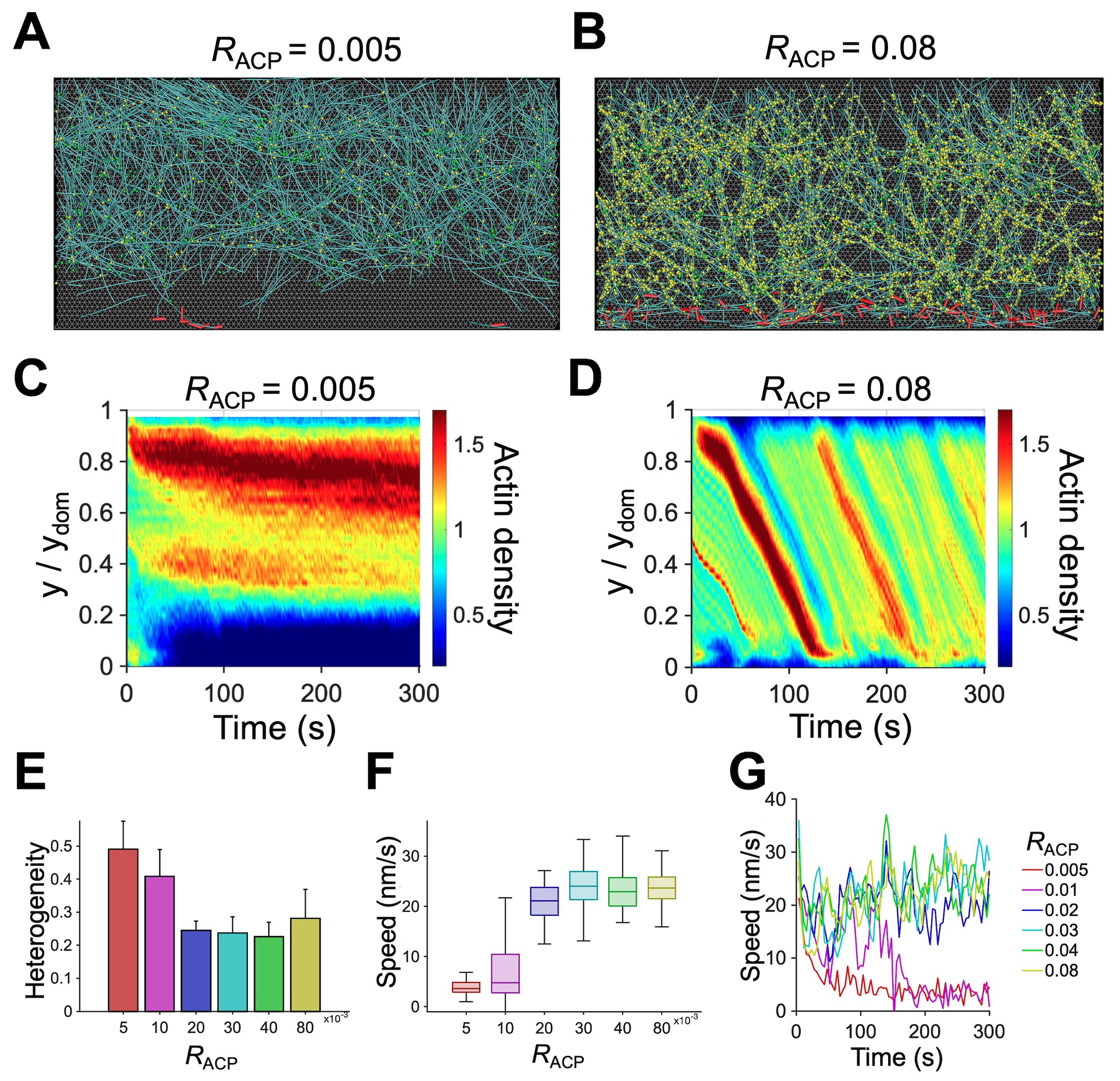

Supplement: S4 Fig — (A, B) Snapshots showing the branched network taken at ~150 s with a lower (0.005) or higher (0.08) RACP relative to that of the reference condition, 0.04. (C, D) Kymographs of actin concentration as a function of y position and time with different RACP. With higher RACP, the network showed more homogeneous morphology and a continuous flow. (E) Heterogeneity of the network quantified as a coefficient of variation in actin density in the y direction. (F) Retrograde flow speed with different RACP. With low RACP values, network heterogeneity was higher, and flow speed was slower. (G) Time evolution of retrograde flow speed. (TIFF) [file pcbi.1013572.s004.tiff]

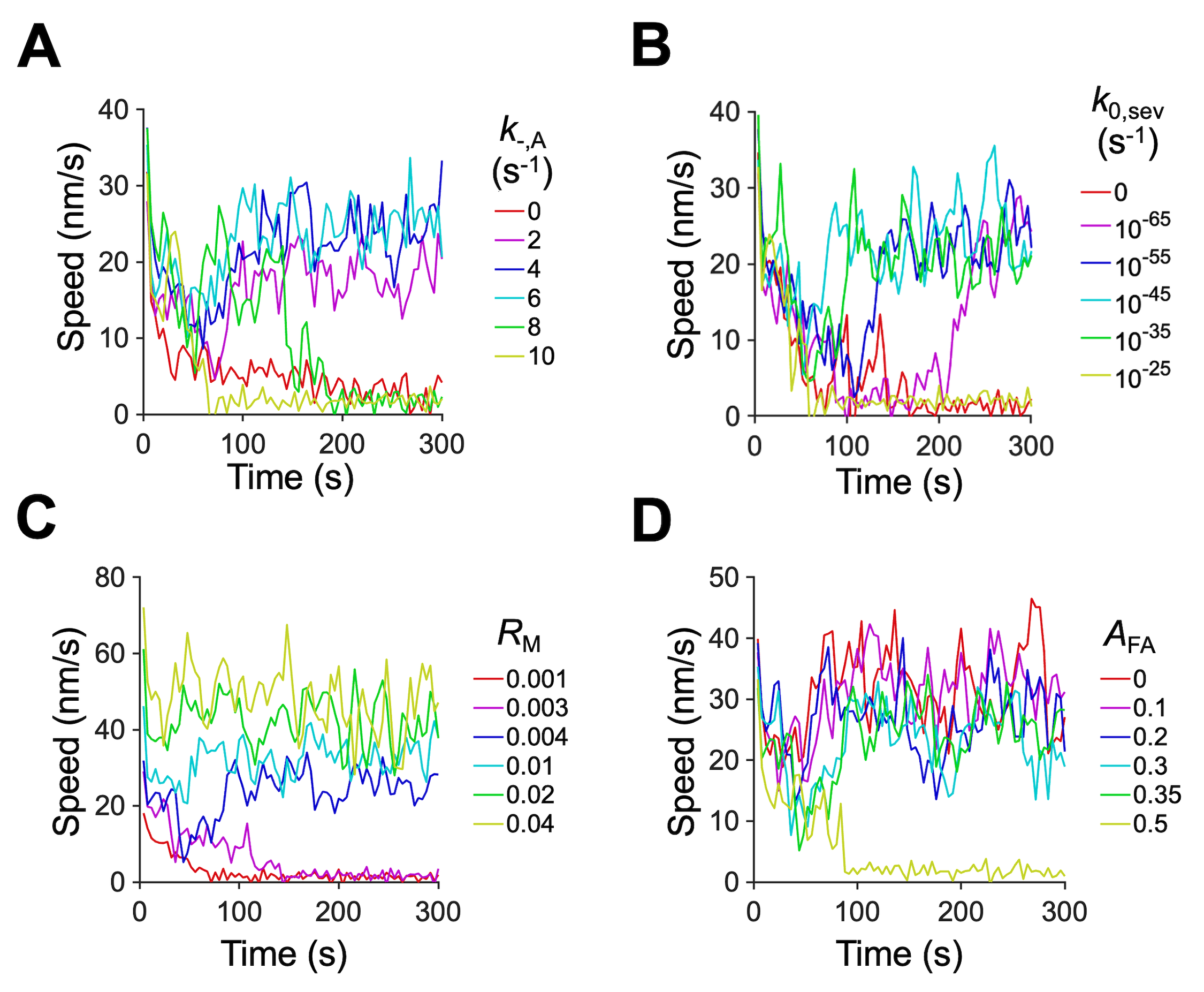

Supplement: S5 Fig — (A) Actin depolymerization rate (k-,A). (B) Actin severing rate constant (k0,sev). (C) Motor density (RM). (D) Relative size of FA region (AFA). (TIFF) [file pcbi.1013572.s005.tiff]

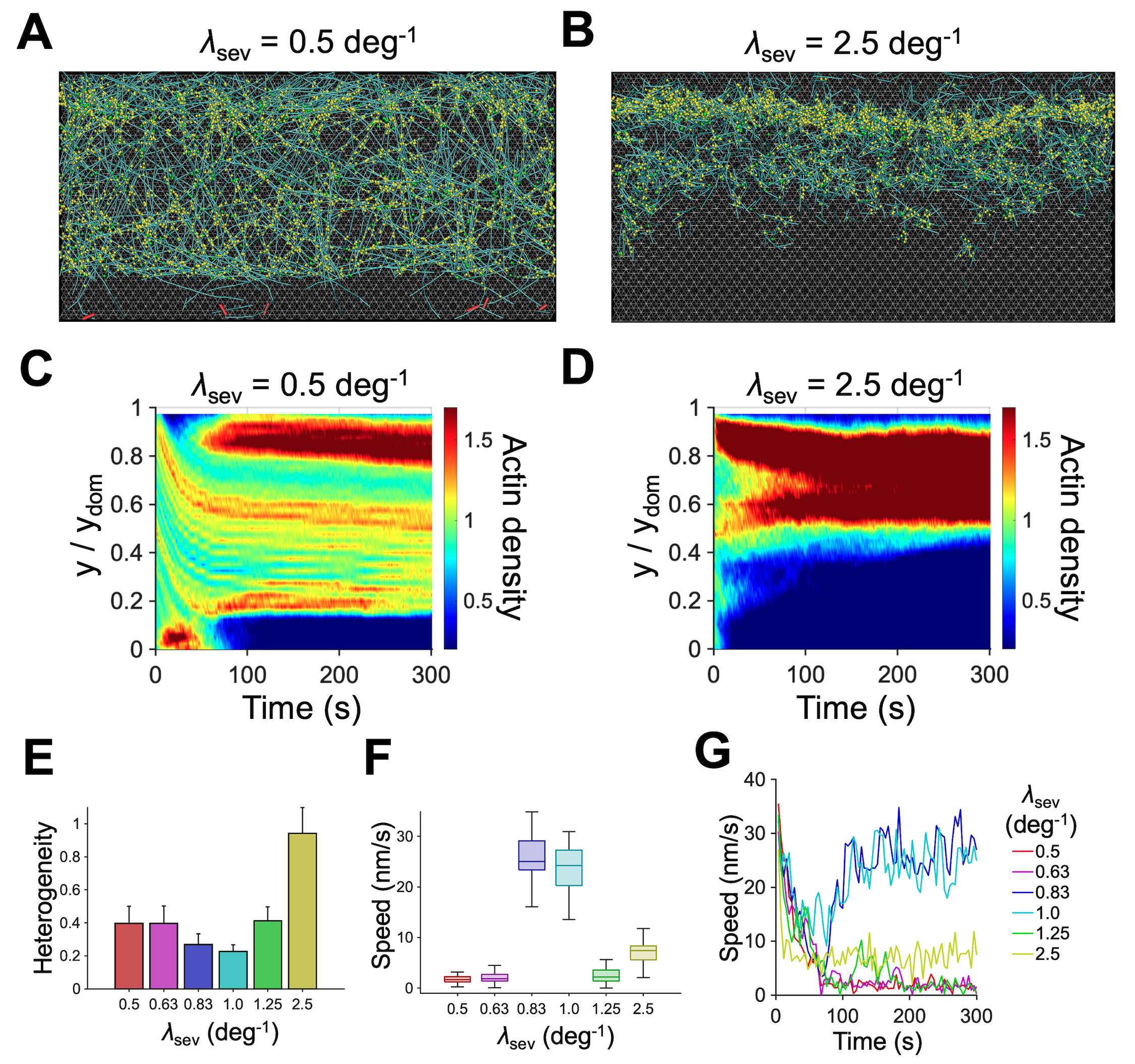

Supplement: S6 Fig — (A, B) Snapshots of the branched network taken at ~150 s with lower (0.5 deg-1) or higher (2.5 deg-1) λsev relative to that of the reference condition, 1.0 deg-1. (C, D) Kymographs of actin concentration as a function of y position and time with different λsev. (E) Heterogeneity of the network quantified as a coefficient of variation in actin density in the y direction. (F) Retrograde flow speed with different λsev. Cases with intermediate λsev reached a steady state with faster flow speed and homogenous network. (G) Time evolution of retrograde flow speed. (TIFF) [file pcbi.1013572.s006.tiff]

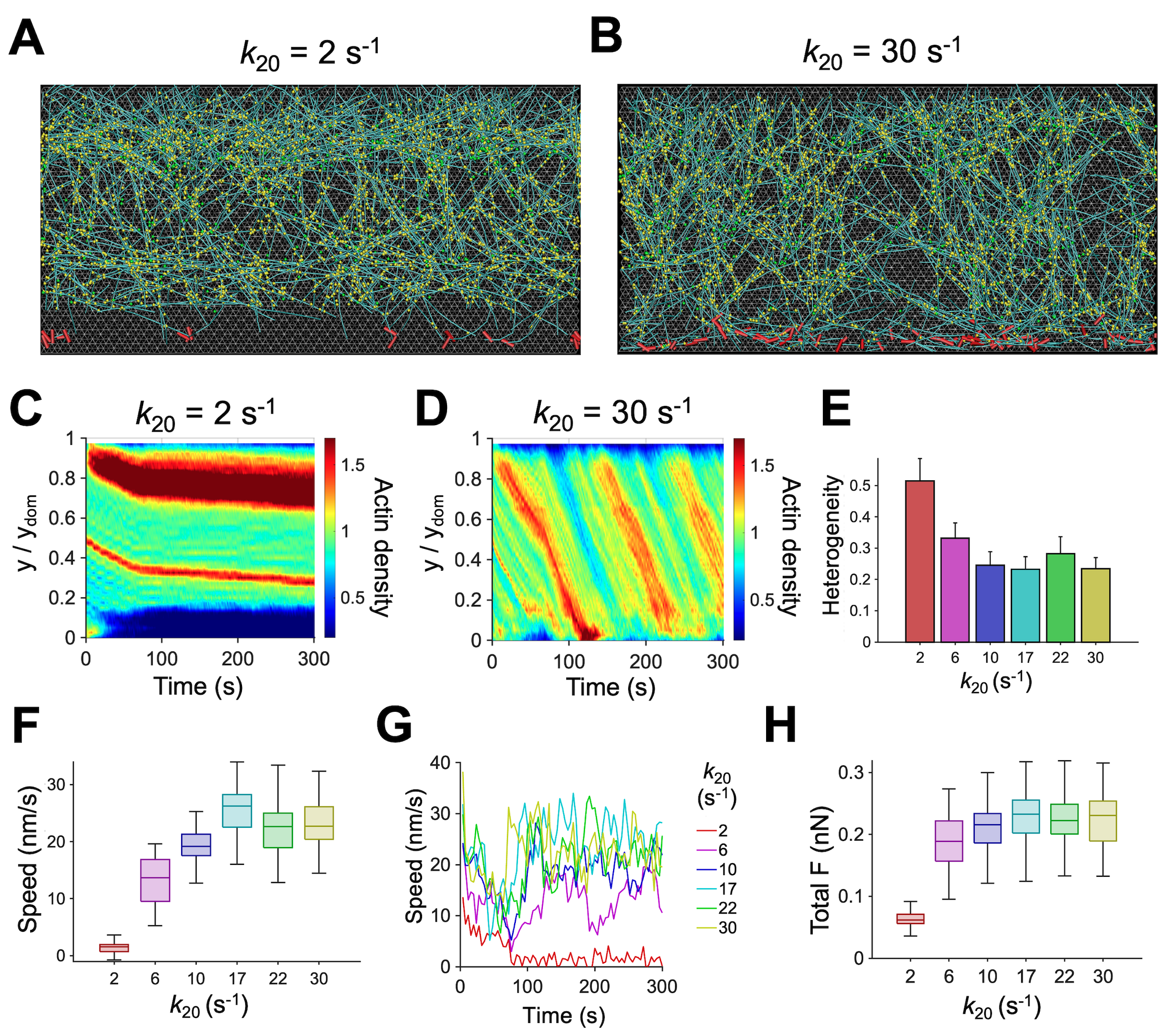

Supplement: S7 Fig — With higher k20, walking speed tends to be higher. (A, B) Snapshots of the branched network taken at ~150 s with lower (2 s-1) or higher (30 s-1) k20 relative to that of the reference condition, 17 s-1. (C, D) Kymographs of averaged actin concentration as a function of y position and time with lower or higher k20. With the lowest k20, the network was disconnected from motors due to excessively slow flow speed. (E) Heterogeneity of the network quantified as a coefficient of variation in actin density in the y direction. (F) Retrograde flow speed with different k20. (G) Time evolution of retrograde flow speed. (H) Total force acting on the substrate by the network with different k20. Flow speed and total substrate force were proportional to k20 at k20 ≤ 17 s-1 and reached a plateau at k20 > 17 s-1. (TIFF) [file pcbi.1013572.s007.tiff]
