# Supplementary material for: The mechanism of dynamic steady states in lamellipodia
Source: PLoS Comput Biol. 2025 Oct 7;21(10):e1013572. doi: 10.1371/journal.pcbi.1013572 (PMC12517506; doi:10.1371/journal.pcbi.1013572)
Supplement: S1 Table — Some of the parameters with references were determined using experimental measurements. Other parameters were determined based on our prior studies [16,77,79]. Each of key parameters tested in this study was tested before the reference case was identified. (PDF) [file pcbi.1013572.s008.pdf]

S1 Table. List of parameters used for the lamellipodium model. Some of the parameters with references were determined using experimental measurements. Other parameters were determined based on our prior studies (1–3). Each of key parameters tested in this study was tested before the reference case was identified.

Symbol	Definition	Value
$r_{0,A}$	Length of an actin segment	1.4×10^{-7} [m]
$r_{c,A}$	Diameter of an actin segment	7.0×10^{-9} [m] (4)
$\theta_{0,A}$	Bending angle formed by adjacent actin segments	0 [rad]
$\kappa_{s,A}$	Extensional stiffness of F-actin	1.69×10^{-2} [N/m]
$\kappa_{b,A}$	Bending stiffness of F-actin	2.64×10^{-19} [N·m] (5)
$r_{0,ACP}$	Length of an ACP segment	2.35×10^{-8} [m] [81]
$r_{c,ACP}$	Diameter of an ACP segment	1.0×10^{-8} [m]
$\theta_{0,ACP}$	Bending angle formed by two ACP segments	0 [rad]
$\kappa_{s,ACP}$	Extensional stiffness of ACP	2.0×10^{-3} [N/m]
$\kappa_{b,ACP}$	Bending stiffness of ACP	1.04×10^{-19} [N·m]
$r_{0,Arp2/3}$	Length of an Arp2/3 segment	3.85×10^{-8} [m] (6)
$r_{c,Arp2/3}$	Diameter of an Arp2/3 segment	1.0×10^{-8} [m]
$\theta_{0,Arp2/3c}$	Bending angle formed by two Arp2/3 segments	0 [rad]
$\theta_{0,Arp2/3m}$	Bending angle between an Arp2/3 segment and a mother filament	1.57 [rad]
$\theta_{0,Arp2/3d}$	Bending angle between an Arp2/3 segment and a daughter filament	0.35 [rad]
$\theta_{0,Arp2/3f}$	Bending angle between mother and daughter filaments	1.22 [rad]
$\phi_{0,Arp2/3}$	Torsion angle between mother and daughter filaments	0 [rad]
$\kappa_{s,Arp2/3}$	Extensional stiffness of Arp2/3 arms	2.0×10^{-3} [N/m]
$\kappa_{b,Arp2/3c}$	Bending stiffness for $\theta_{Arp2/3c}$	1.0×10^{-19} [N·m]
$\kappa_{b,Arp2/3m}$	Bending stiffness for $\theta_{Arp2/3m}$	1.0×10^{-18} [N·m]
$\kappa_{b,Arp2/3d}$	Bending stiffness for $\theta_{Arp2/3d}$	1.0×10^{-18} [N·m]
$\kappa_{b,Arp2/3f}$	Bending stiffness for $\theta_{Arp2/3f}$	1.0×10^{-18} [N·m]
$\kappa_{t,Arp2/3}$	Torsional stiffness of Arp2/3	1.0×10^{-18} [N·m]
$r_{0,MB}$	Length of a motor backbone segment	4.2×10^{-8} [m]
$r_{0,M1}$	Length of the transverse spring for a motor arm	1.35×10^{-8} [m]
$r_{0,M2}$	Length of the longitudinal spring for a motor arm	0 [m]
$r_{c,M}$	Diameter of a motor arm	1.0×10^{-8} [m]
$\theta_{0,M}$	Bending angle formed by motor backbone segments	0 [rad]
$\kappa_{s,MB}$	Extensional stiffness of a motor backbone	1.69×10^{-2} [N/m]
$\kappa_{s,M1}$	Extensional stiffness for the transverse spring for a motor arm	1.0×10^{-3} [N/m]
$\kappa_{s,M2}$	Extensional stiffness for the longitudinal spring for a motor arm	1.0×10^{-3} [N/m]
$\kappa_{b,M}$	Bending stiffness of a motor backbone	5.07×10^{-18} [N·m]
$\kappa_{r,A}$	Strength of repulsive force between F-actins	1.69×10^{-3} [N/m]
N_h	Number of heads represented by a motor arm	4
N_a	Number of arms per motor	8
k_{20}	Unbinding rate of motor head	17.14 [s^{-1}]*
$k_{n,A}$	De novo nucleation rate of actin	2.0×10^{-7} [$\mu M^{-1}s^{-1}$]

$k_{+,A}$	Polymerization rate of actin at the barbed end	12 [$\mu M^{-1}s^{-1}$]*
$k_{-,A}$	Depolymerization rate of actin at the pointed end	6 [s^{-1}]*
$k_{+,Arp2/3}$	Binding rate of Arp2/3	20 [$\mu M^{-1}s^{-1}$]
$k_{+,ACP}$	Binding rate of ACP	100 [$\mu M^{-1}s^{-1}$]
C_A	Actin concentration	250 [μM]
$R_{Arp2/3}$	Ratio of Arp2/3 concentration to C_A	0.01*
R_{ACP}	Ratio of ACP concentration to C_A	0.04*
R_M	Ratio of motor concentration to C_A	0.004*
$k_{0,sev}$	Severing rate constant	10^{-45} [s^{-1}]*
λ_{sev}	Sensitivity to F-actin angle	1.0 [deg^{-1}]*
A_{FA}	Size of focal adhesion region relative to domain size	0.35*
Δt	Time step	1.15×10^{-5} [s]
μ	Viscosity of surrounding medium	8.6×10^{-1} [kg/m·s]
k_BT	Thermal energy	4.142×10^{-21} [J]
$\kappa_{s,sub}$	Extensional stiffness of a substrate	1.0×10^{-4} [N/m]
$\kappa_{b,sub}$	Bending stiffness of a substrate	2.4×10^{-20} [Nm]
$r_{0,sub}$	Length of a substrate chain	5.0×10^{-8} [m]
$\theta_{0,sub}$	Bending angle formed by substrate chains	1.05 [rad]
$\kappa_{s,C}$	Extensional stiffness of links	1.0×10^{-3} [N/m]
$k_{+,C}$	Binding rate of a link	1.0 [s^{-1}]
$k_{u,C}^{0}$	Zero-force dissociation rate constant of a links	0.14 [s^{-1}]
$x_{u,C}$	Sensitivity of the link dissociation rate to applied force	2.9×10^{-9} [m]

References

1. Mair DB, Elmasli C, Kim JH, Barreto AD, Ding S, Gu L, et al. The Arp2/3 complex enhances cell migration on elastic substrates. *Mol Biol Cell*. 2023;34(1):ar67. doi:10.1091/mbc.E22-06-0243

2. Muresan CG, Machesky LM, et al. F-actin architecture determines constraints on myosin thick filament motion. *Nat Commun*. 2022;13:7008. doi:10.1038/s41467-022-34767-7

3. Jung W, Murrell MP, Kim T. F-actin cross-linking enhances the stability of force generation in disordered actomyosin networks. *Comput Part Mech*. 2015;2(3):317–27. doi:10.1007/s40571-015-0052-9

4. Meyer RK, Aebi U. Bundling of actin filaments by alpha-actinin depends on its molecular length. *J Cell Biol*. 1990;110(6):2013–24. doi:10.1083/jcb.110.6.2013

5. Isambert H, Venier P, Maggs AC, Fattoum A, Kassab R, Pantaloni D, et al. Flexibility of actin filaments derived from thermal fluctuations. *J Biol Chem*. 1995;270(19):11437–44. doi:10.1074/jbc.270.19.11437

6. Meyer RK, Aebi U. Bundling of actin filaments by alpha-actinin depends on its molecular length. *J Cell Biol*. 1990;110(6):2013–24. doi:10.1083/jcb.110.6.2013
